# Spin-orbit density wave induced hidden topological order in URu_2_Si_2_

**DOI:** 10.1038/srep00596

**Published:** 2012-08-22

**Authors:** Tanmoy Das

**Affiliations:** 1 Theoretical Division, Los Alamos National Laboratory, Los Alamos, NM 87545, USA; 2 Physics Department, Northeastern University, Boston, MA 02115, USA

## Abstract

The conventional order parameters in quantum matters are often characterized by ‘spontaneous’ broken symmetries. However, sometimes the broken symmetries may blend with the invariant symmetries to lead to mysterious emergent phases. The heavy fermion metal URu_2_Si_2_ is one such example, where the order parameter responsible for a second-order phase transition at *T_h_* = 17.5 K has remained a long-standing mystery. Here we propose via *ab-initio* calculation and effective model that a novel spin-orbit density wave in the *f*-states is responsible for the hidden-order phase in URu_2_Si_2_. The staggered spin-orbit order spontaneously breaks rotational, and translational symmetries while time-reversal symmetry remains intact. Thus it is immune to pressure, but can be destroyed by magnetic field even at *T* = 0 K, that means at a quantum critical point. We compute topological index of the order parameter to show that the hidden order is topologically invariant. Finally, some verifiable predictions are presented.

Most states or phases of matter can be described by local order parameters and the associated broken symmetries in the spin, charge, orbital or momentum channel. However, recent discoveries of quantum Hall states^1^, and topological insulators^2^,^3^ have revamped this conventional view. It has been realized^1^,^2^,^3^,^4^ that systems with combined time-reversal (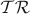) symmetry and large spin-orbit (SO) coupling can host new states of matter which are distinguished by topological quantum numbers of the bulk band structure rather than spontaneously broken symmetries. Subsequently, more such distinct phases have been proposed in the family of topological Mott insulators^5^, topological Kondo insulators^6^, topological antiferromagnetic insulators^7^. In the latter cases, the combined many-body physics and 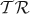 symmetry governs topologically protected quantum phases. Encouraged by these breakthrough developments, we search for analogous exotic phases in the heavy fermion metal URu_2_Si_2_, whose low-energy *f* states accommodate 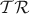 and strong SO coupling. This compound also naturally hosts diverse quantum mechanical phases including Kondo physics, large moment antiferromagnetism (LMAF), mysterious ‘hidden-order’ (HO) state, and superconductivity^8^.

In URu_2_Si_2_ the screening of *f*-electrons due to the Kondo effect begins at relatively high temperatures, ushering the system into a heavy fermion metal at low-temperature^9^. Below *T_h_* = 17.5 K, it enters into the HO state via a second-order phase transition characterized by sharp discontinuities in numerous bulk properties^10^,^11^,^12^,^13^. The accompanying gap is opened both in the electronic structure^9^,^14^,^15^,^16^ as well as in the magnetic excitation spectrum^17^, suggesting the formation of an itinerant magnetic order at this temperature. However, the associated tiny moment (~ 0.03*µ_B_*) cannot account for the large (about 24%) entropy release^18^ and other sharp thermodynamic^10^,^11^ and transport anomalies^12^,^13^ during the transition. Furthermore, very different evolutions of the HO parameter and the magnetic moment as a function of both magnetic field^19^,^20^ and pressure^21^,^22^ rule out a possible magnetic origin of the HO phase in this system. Any compelling evidence for other charge, orbital or structural ordering has also not been obtained^23^. Existing theories include multiple spin correlator^24^, Jahn-Teller distortions^25^, unconventional spin-density wave^26^,^27^, antiferromagnetic fluctuation^28^, orbital order^20^, helicity order^29^, staggered quadrupole moment^30^, octupolar moment^31^, hexadecapolar order^32^, linear antiferromagnetic order^33^, incommensurate hybridization wave^34^, spin nematic order^35^, modulated spin liquid^36^, *j*-*j* fluctuations^37^, unscreened Anderson lattice model^38^, among others^8^. However, a general consensus for the microscopic origin of the HO parameter has not yet been attained.

Formulating the correct model for the HO state requires the knowledge of the broken symmetries and the associated electronic degrees of freedom that are active during this transition. A recent torque measurement on high quality single crystal sample reveals that the four-fold rotational symmetry of the crystal becomes spontaneously broken^23^ at the onset of the HO state. Furthermore, several momentum-resolved spectroscopic data unambiguously indicate the presence of a translational symmetry breaking at a longitudinal incommensurate wavevector ***Q****_h_* = (1±0.4, 0, 0)^14^,^16^,^18^,^39^. [Previous first-principle calculation has demonstrated that an accompanying commensurate wavevector ***Q***_2_ = (1, 0, 0) might be responsible for the LMAF phase^33^, which is separated from the HO state via a first order phase transition^8^,^19^,^20^,^21^,^22^. As it is often unlikely to have two phases of same broken symmetry but separated by a phase boundary, we expect that LMAF and HO phases are different.] In general, the order parameter that emerges due to a broken symmetry relies incipiently on the good quantum number and symmetry properties of the ‘parent’ or non-interacting Hamiltonian. In case of URu_2_Si_2_, spin and orbital are not the good quantum numbers, rather the presence of the SO coupling renders the total angular momentum to become the good quantum number. Therefore, *SU*(2) symmetry can not be defined for spin or orbital alone, and the ‘parent’ Hamiltonian has to be defined in 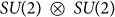 representation. The ‘parent’ Hamiltonian also accommodate other symmetries coming from its crystal, wavefunction properties which we desire to incorporate to formulate the HO parameter.

## Results

### Ab-initio band structure

**Figure 1:**
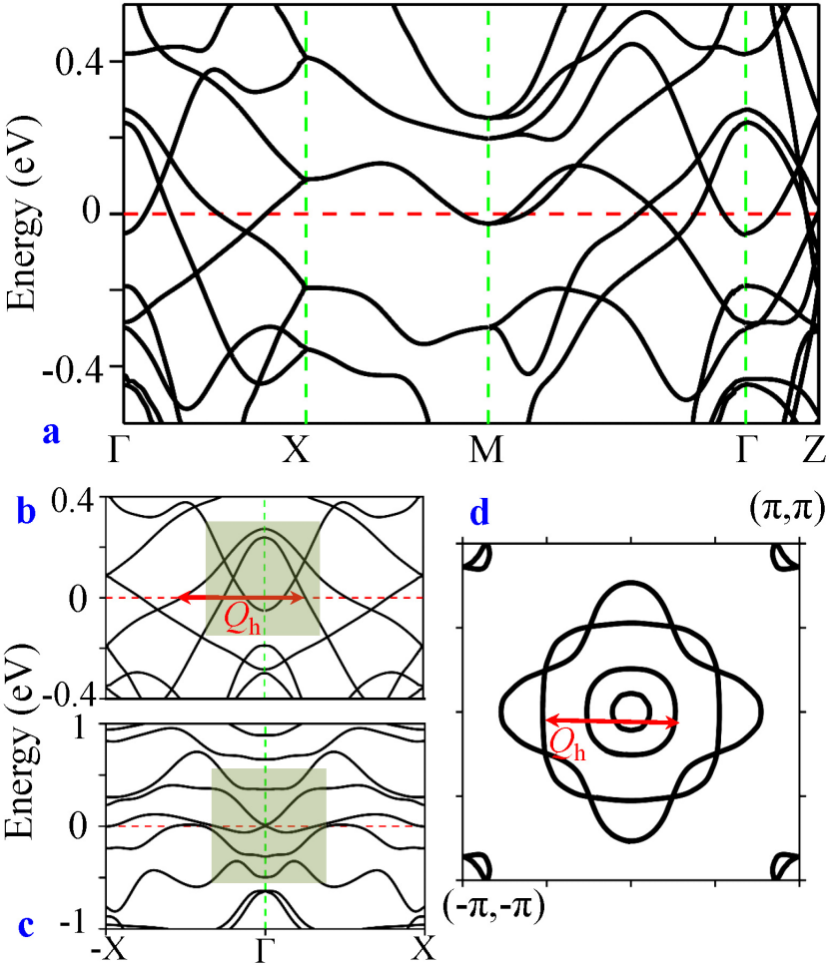
*Ab initio* band structure and Fermi surface of URu_2_Si_2_. (a) Computed non-interacting energy dispersions of URu_2_Si_2_, using Wien2K software^40^,^41^, are presented along Γ(0,0,0), X(*π*,0,0), M(*π*,*π*,0), and Z(0,0,*π*) directions. The band structure is consistent with the previous full potential local orbitals (FPLO) and full potential linearized augmented plane wave (FPLAPW) calculations in the paramagnetic state^33^. The low-energy dispersions along Γ-X is expanded in (b) and contrasted with the same but without the SO coupling in (c). The FS in the *k_z_* = 0 plane is shown in (d). The red arrow dictates the FS ‘hot-spot’ that emerges after including SO coupling.

In order to find out the symmetry properties of the low-lying states, we begin with investigating the *ab-initio* ‘parent’ band dispersion and the FS of URu_2_Si_2_^40^,^41^ in Fig. 1. The electronic structure in the vicinity of the Fermi level (*E_F_*) (±0.2 eV) is dominated by the 5*f* states of U atom in the entire Brillouin zone^14^,^15^,^16^,^33^,^39^,^42^. Owing to the SO coupling and the tetragonal symmetry, the 5*f* states split into the octet 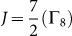 states and the sextet 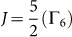 states^43^. URu_2_Si_2_ follows a typical band progression in which the Γ_8_ bands are pushed upward to the empty states while the Γ_6_ states drop to the vicinity of *E_F_*. The corresponding FS in Fig. 1d reveals that an even number of anti-crossing features occurs precisely at the intersection between two oppositely dispersing conducting sheets. Unlike in topological insulators^3^,^4^, the departure of the band crossing points from the 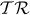-invariant momenta here precludes the opening of an inverted band gap at the crossings^2^, and Dirac-cones crop up with Kramer's degeneracy in the bulk states. Therefore, URu_2_Si_2_ is an intrinsically trivial topological metal above the HO transition temperature.

**Figure 2:**
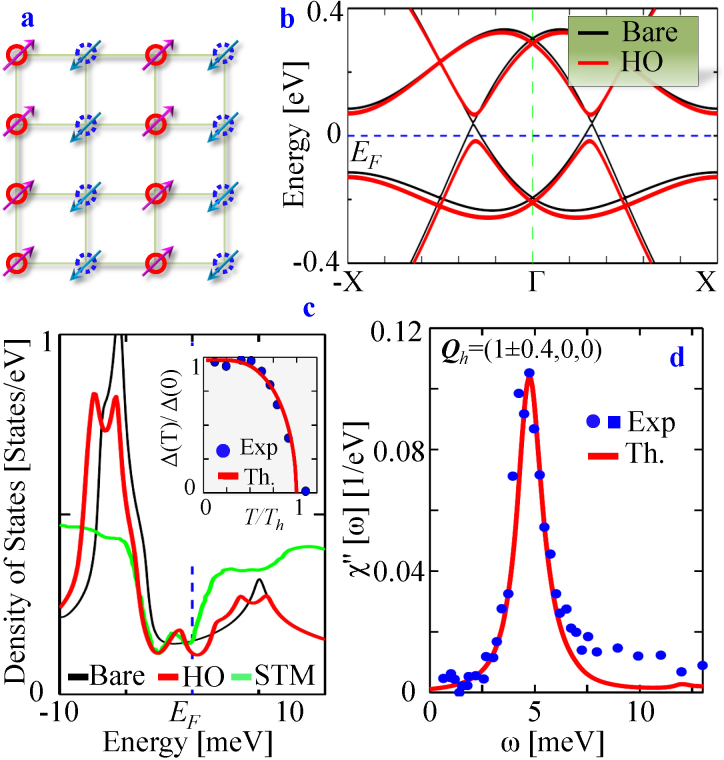
Spin-orbit density wave and the hidden-order gap opening. (a) A typical form of the staggered SO order is schematically described for an illustrative case of commensurate wavevector. The solid and dashed circles encode two opposite orbitals, *τ* = ±, where the associated arrows depict their ‘pseudospins’ *σ*. Both *τ* and *σ*, representing orbital and spin respectively, individually break 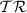 symmetry, while their product remains 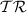 invariant. (b) Model dispersions of the 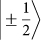, and 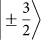 subbands plotted along the axial direction. Black and red lines give dispersion before and after including the HO gap, respectively. An artificially large value of Δ = 50 meV is chosen here to clearly explicate the momentum dependence of the modulated SO gap opening. (c) Modifications of DOS upon entering into the HO phase are compared with measured DOS in the STM experiment (green line)^14^. Note that the experimental data is subtracted from the background spectrum at *T*>*T_h_*, which helps highlight the appearance of multiple structures in the DOS spectrum at the HO state. Here the gap magnitude Δ(0) = 5 meV, obtained at a coupling strength of *g* = 27 meV, see SI. *Inset:* The self-consistent value of Δ(*T*) exhibits the mean-field behavior of the HO gap, in consistent with experiments^9^. We obtain *T_h_* = 22 K which is larger than the experimental value of *T_h_* = 17.5 K. However, recently it has been pointed out that there exists a ‘pseudogap’ above the HO state^58^, which presumably reduces the mean-field temperature scale. (d) RPA result of SO correlation function at *g* = 28.4 meV shows a resonance peak at *ω_Q_* = 4.7 meV at *Q_h_*, in good agreement with experimental data^18^,^59^.

The SO interaction introduces two prominent FS instabilities at *Q*_2_ = (1, 0, 0) and at *Q_h_* = (1 ± 0.4, 0, 0). The commensurate wavevector *Q*_2_ occurs between same orbital. Therefore, if this instability induces a gap opening, it has to be in the spin-channel, which is prohibited by 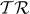 symmetry and strong SO coupling. We argue (see Supplementary Information (SI) for details), in accordance with an earlier calculation^33^, that this instability is responsible for the LMAF phase. On the other hand, the incommensurate one, *Q_h_*, occurs between two different orbitals, and can open a gap if a symmetry between these orbitals and spins are spontaneously broken together. In other word, since SO coupling is strong in this system, individual spin- or orbital-orderings are unlikely to form unless interaction can overcome the SO coupling strength. On the other hand, a SO entangled order parameter in the two-particle channel can collectively propagate with alternating sign in the total angular momentum at the wavelength determined by the modulation vector. This is the guiding instability that drives spontaneous rotational symmetry breaking, while the 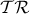 symmetry remains intact (see Fig. 2a). This is because, both *SU*(2) groups for spin and orbital separately are odd under 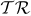, but their product 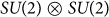 becomes even. As the parent state is not a non-trivial topological phase, a gap is opened to lift the FS instability.

### Low-energy effective model

Motivated by the above-mentioned experimental results and band structure symmetry properties, we formulate a simple and unified model by using the theory of invariants^44^. We restrict our discussion to the low-lying Γ_6_ bands and neglect the unfilled Γ_8_ bands. Due to *j*-*j* SO coupling and 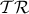 symmetry, the Γ_6_ atomic states consist of three doublets, characterized by up and down ‘pseudospins’: 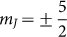, 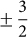, 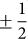, where *m_J_* is the *z* component of *J*. On entering into the HO state, the FS instability commences in between the two doubly degenerate 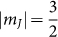 and 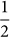 states only^33^,^35^. If no other symmetry is broken, the degenerate 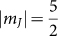 state remains unaltered in the HO state^44^, and hence they are not considered in our model Hamiltonian. Throughout this paper, we consistently use two indices: orbital index 
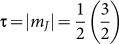
, and ‘pseudospin’ *σ* = ↑(+), ↓(−). In this notation, we consider the ‘pseudospinor’ field 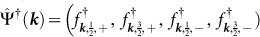, where 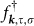 is the creation operator for an electron in the orbital 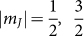 with momentum ***k*** and ‘pseudospin’ *σ*.

The representation of the symmetry operations that belongs to the *D*_4*h*_ symmetry of the URu_2_Si_2_ crystal structure is: 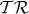 symmetry, inversion symmetry 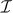, four-fold rotational symmetry 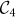, and the two reflection symmetries 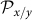. The SO *f*-state of actinides is invariant under all symmetries except the mirror reflection, which in fact allows the formation of the SO density wave into a finite gap in the HO state (see *SI*). On the basis of these symmetry considerations, it is possible to deduce the general form of the non-interacting Hamiltonian as: 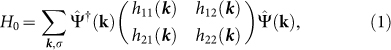

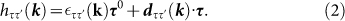
Here, *τ^µ^* (*µ* 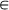 0, *x*, *y*, *z*) depict the 2D Pauli matrices in the orbital space and *τ*^0^ is the identity matrix (***σ****^µ^* matrices will be used later to define the spin space). The 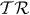 invariance requires that 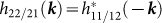. Under 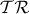 and 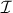, the symmetry of 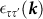 and 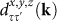 must complement to their corresponding identity and Pauli Matrix counterparts, respectively. Hence we obtain the Slater-Koster hopping terms as: 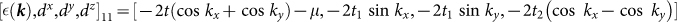 and 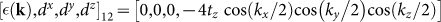. The obtained values of the tight-binding hopping parameters as (*t*, *t*_1_, *t*_2_, *t_z_*) = −(−45,45,50,−25) in meV. The above Hamiltonian can be solved analytically which gives rise to four SO-split energy dispersions as 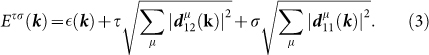
Here *σ* = ± and *τ* = ± become band indices. An important difference of the present Hamiltonian with that of bulk topological insulators^3^ or quantum spin-Hall systems^1^ is the absence of a mass or gap parameter in the former case. The computed non-interacting bands are plotted in Fig. 2b, which exhibit several Dirac points along the high-symmetry lines. Focusing on the Dirac point close to *E_F_*, we find that it occurs at the crossing between bands *E*^+−^ and *E*^−+^, demonstrating that it hosts four-fold Kramer's degeneracy (two orbitals and two spins). Therefore, lifting this degeneracy requires the presence of a SO order parameter. However, it is important to note that the gap opening at the Dirac point is not a manifestation of the presence of degeneracy at it, but a consequence of the SO density wave caused by FS instability.

### SO density wave induced HO

The ‘hot-spot’ ***Q****_h_* divides the unit cell into a reduced ‘SO Brillouin zone’ in which we can define the Nambu operator in the usual way 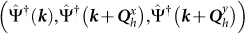. In this notation, the SO density wave (SODW) interaction term can be written in general as 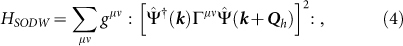
where *µ, ν* ∈ {0, *x, y, z*}. The symbol :: represents normal ordering. Here *g* is the contact coupling interaction arising from screened interorbital Coulomb term embedded in Hund's coupling parameter, and 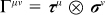, ***τ*** and ***σ*** represent Pauli matrices in orbital and spin basis, respectively. Absorbing *g* and Γ into one term we define the mean-field order parameter 
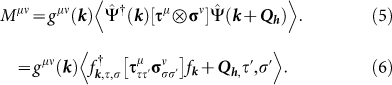
Here *τ*, *τ*′ and *σ*, *σ*′ (not in bold font) are the components of the ***τ****^µ^* and ***σ****^ν^* matrices, respectively. Without any loss of generality we fix the spin orientation along *z*-directions (*ν* = *z*). Therefore, we drop the index *ν* henceforth. Furthermore we define the gap vector as 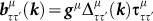, where we split the interaction term *g*(***k***) into a constant onsite term and the dimensionless order parameter Δ(***k***). With these substitutions, we obtain the final result for the order parameter as 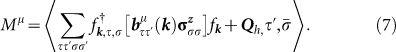
Eq. 7 admits a plethora of order parameters related to the SO density wave formations which break symmetry in different ways. Among them, we rule out those parameters which render gapless states by using the symmetry arguments (see *SI*): All four order parameters obey 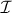 symmetry, while only *M^y^* term is even under 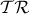, because it is the product of two odd terms ***τ****^y^* and ***σ*** (we drop the superscript ‘*y*’ henceforth). This is the only term which commences a finite gap opening if the translational or rotational symmetry is spontaneously broken. We have shown in *SI* that there exists a considerably large parameter space of coupling constant ‘*g*’ where this order parameter dominates.

Eq. 7 implies that spin and orbital orderings occur simultaneously along the ‘hot-spot’ direction ***Q***_h_, as illustrated in Fig. 2a. It propagates along 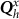 or 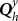 directions with alternating signs (particle-hole pairs) to commence a SO density wave. The resulting Hamiltonian breaks the four-fold rotational symmetry down to a two-fold one 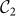, and gives rise to a so-called spin-orbit ‘smectic’ state which breaks both translational and *C*_4_ symmetry^45^. The present 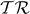 invariant SO order parameter is inherently distinct from any spin or orbital or even interorbital spin-density wave order which break 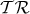 symmetry. This criterion also rules out any similarly between our present SO smectic state with the spin-nematic phase^35^ or spin-liquid state^36^. Furthermore, the present order parameter is different from 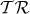 invariant ‘hybridization wave’ (between *f* and *d* orbitals of same spin), or charge density wave or others^30^,^32^, as SO order involves flipping of both orbital (between split *f* orbitals that belong to Γ_6_ symmetry) and spin simultaneously. Taking into account the band-structure information that ***Q*_h_** represents the interband nesting, it is instructive to focus on only *b*_12_(*k*) component (thus the subscript ‘12’ is eliminated hereafter). Therefore, the SO density wave does not introduce a spin or orbital moment, but a polarization in the total angular momentum *δm_J_* = ±2 [for the ordering between 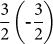 and 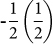].

The ***b*** vector belongs to the same irreducible point group representation, *E_g_*, of the crystal with odd parity, and can be defined by |***b***(***k***)| = 2*ig*Δ*^x^* sin *k_x_a*, or 2*ig*Δ*^y^* sin *k_y_a* for the wavevectors 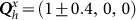, or 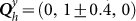, respectively. The mean-field Hamiltonian for the HO state within an effective two band model reduces to the general form *H_MF_* = *H*_0_+*H_SODW_*, where the particle-hole coupling term is 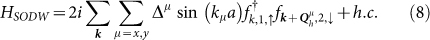
In the Nambu representation, it is obvious that the HO term merely adds a mass term to the 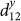 term defined above. At the band-crossing points located where 
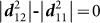
, a gap opens by the value of |***b*(*k***)|^2^. Figure 2 demonstrates the development of the quasi-particle structure in the HO state. The band progression and the associated gap opening is fully consistent with the angle-resolved photoemission spectroscopy (ARPES) observations^15^,^16^. The scanning tunneling microscopy and spectroscopic (STM/S)^9^,^14^ fingerprints of the gap opening in the density of state (DOS) is also described nicely within our calculations, see Fig. 2c.

## Discussion

The SO moment is 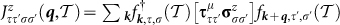, where 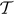 is imaginary time. Introducing simplified indices *α, β* = *ττ*′*σσ*′, the correlation function of 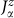 vector can be defined as 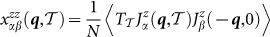, where 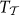 is normal time-ordering. Our numerical calculation of the 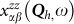 within random-phase approximation (RPA) yields an inelastic neutron scattering (INS) mode with enhanced intensity at ***Q*_h_** near *ω_Q_* ~ 4.7 meV below *T_h_* as shown in Fig. 1d. INS data (symbols) at a slightly large momentum agrees well with our calculation, however, a polarized INS measurement will be of considerable value to distinguish our proposed 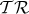 invariant mode from any spin-flip and elastic background^46^. A *T*-dependent study of the INS mode also reveals that this mode becomes strongly enhanced at *Q_h_* rather than at the commensurate one below *T_h_*^39^.

One way to characterize the nature of a phase transition is to determine the temperature evolution of the gap value. Our computed self-consistent values of the mean-field gap Δ(*T*) agree well with the extracted gap values from the STM spectra^9^ [see *inset* to Fig. 2c]. In general, the entropy loss at a mean-field transition is given by^20^ 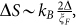, where Δ is the HO gap and *ξ_F_* is the Fermi energy of the gapped state. At HO the Fermi energy 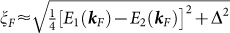, where the two linearly dispersive bands near the Fermi level yields 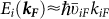. Using the measured Sommerfield coefficient *γ* = 180 mJmol^−1^K^−2^, compared to its linear expansion of *γ*_0_ = 50 mJmol^−1^K^−2^, we obtain the mass renormalization factor *Z*^−1^ = *γ*/*γ*_0_ = 3.6. This gives 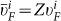. For the two bands that participate in the HO gap opening, we get 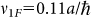 in eV at *k*_1*F*_ = 0.5*π*/*a* and 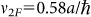 in eV at *k*_2*F*_ = 0.3*π*/*a* from Fig. 1a. Using the experimental value of Δ = 5 meV^9^,^14^, we obtain Δ*S*~ 0.28*k_B_* ln 2, which is close the experimental value of 0.3*k_B_* ln 2^10^.

We now evaluate the topological invariant index of interacting Hamiltonian in Eq. 8 to demonstrate that HO gap opening in URu_2_Si_2_ also induces topological phase transition. To characterize the topological phenomena, we recall the Fu-Kane classification scheme^2^ which implies that if a time-reversal invariant system possess an odd value of *Z*_2_ invariant index, the system is guaranteed to be topologically non-trivial. *Z*_2_ index is evaluated by the time-reversal invariant index *ν_i_* = ±1, if defined, for all filled bands as *Z*_2_ = *ν*_1_*ν*_2_…*ν_n_*, where *n* is the total number of orbitals in the Fermi sea. A more efficient method of determining the topological phase is called the adiabatic transformation scheme used earlier in realizing a large class of topological systems, especially when *Z*_2_ calculation is difficult^47^. In this method, the non-trivial topological phase of a system can be realized by comparing its band-progression with respect to an equivalent trivial topological system. URu_2_Si_2_ is topologically trivial above the HO state, i.e. 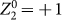. The gap opening makes the top of the valence band (odd parity) to drop below *E_F_* as shown in Fig. 2b. Thereby, an odd parity gained in the occupied level endows the system to a non-trivial topological metal. To see that we evaluate the topological index for the HO term as *ν_ho_* = *∫ d**k***Ω(***k***), where the corresponding Berry curvature can be written in terms of *b*-vector as 
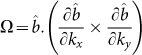
 for each spin with 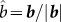. Due to the odd parity symmetry of ***b***, it is easy to show that *ν_ho_* = −1 which makes the total *Z*_2_ value of the HO phase to be 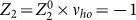, and hence we show that hidden order gapping is a topologically non-trivial phase. The consequence of a topological bulk gap is the presence of surface states^2^,^47^. In our present model, we expect two surface states of opposite spin connecting different orbitals inside the HO gap. As the system is a weak-topological system, the surface states are unlikely to be topologically protected. The SO locking of these states can be probed by ARPES using circular polarized incident photon which will be a definite test of this postulate.

The HO gap is protected from any 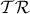 invariant perturbation such as pressure (with sufficient pressure the HO transforms into the LMAF phase), while 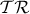 breaking perturbation such as magnetic field will destroy the order. Remarkably, these are the hallmark features of the HO states^23^,^35^,^48^, which find a natural explanation within our SO density wave order scenario. In what follows, the magnetic field will destroy the HO state even at *T* = 0 K, that means at a quantum critical point (QCP) as the HO is a spontaneously broken symmetry phase^49^. However, due to the finite gap opening at the HO state, it requires finite field to destroy the order. The thermodynamical critical field can be obtained from^50^ 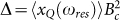, where *B_c_* is the critical field and 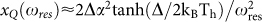 at the resonance mode that develops in the HO state. *α* = *gµ_B_*|〈*Δm_J_*〉| = 2*gµ_B_* and bare *g*-factor *g* = 0.8. Substituting *ω_res_* = 4.7 meV, we get the location of the QCP at *B* ≈ 38 T, which is close the experimental value of *B* = 34 T^48^.

Broken symmetry FS reconstruction leads to enhanced Nernst signal^51^. For the case of broken symmetry SO order, we expect to generate spin-resolved Nernst effect which can be measured in future experiments to verify our proposal^52^.

In summary, we proposed a novel SO density wave order parameter for the HO state in URu_2_Si_2_. Such order parameter is 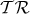 symmetry invariant. We find no fundamental reason why such order parameter cannot develop in other systems in which both electronic correlation and SO of any kind are strong. Some of the possible materials include heavy fermion systems, Iridates^53^, SrTiO_3_ surface states^54^, SrTiO_3_/LiAlO_3_ interface^55^, Half-Heusler topological insulator^47^, and other *d*- and *f*-electron systems with strong SO. In particular, a Rashba-type SO appears due to relativistic effect in two-dimensional electron system yielding helical FSs. In such systems, the FS instability may render similar SO density wave, and the resulting quasiparticle gap opening is observed on the surface state of BiAg_2_ alloys even when the spin-degeneracy remains intact^56^. Furthermore, recent experimental findings of quasiparticle gapping in the surface state of topological insulator due to quantum phase transition even in the absence of time-reversal symmetry breaking can also be interpreted as the development of some sort of spin orbit order^57^.

## Author Contributions

TD has carried out all the calculations, prepared the figures, wrote the paper.

## Supplementary Material

Supplementary Information Detailed formalism
