# Supplementary material for: Spin-orbit density wave induced hidden topological order in URu2Si2
Source: Sci Rep. 2012 Aug 22;2:596. doi: 10.1038/srep00596 (PMC3424526; doi:10.1038/srep00596)
Supplement: Supplementary Information — Detailed formalism [file srep00596-s1.pdf]

Supplementary Information (SI) for "Spin-orbit density wave induced hidden topological order in URu_2Si_2"

Tanmoy Das[1,2]
[1] *Theoretical Division, Los Alamos National Laboratory, Los Alamos, NM, 87545, USA.*
[2] *Physics Department, Northeastern University, Boston, MA, 02115, USA.*
(Dated: August 1, 2012)

In the main text, we have provided an effective two band model which is relevant for the study of hidden-order (HO) gap structure coming from Fermi surface (FS) instability. Here, we expand on how such an effective Hamiltonian is deduced. The spin-orbit splitting state is, in general, studied within either $\boldsymbol{L}.\boldsymbol{S}$ coupling or $j-j$ coupling approaches. In the former case, the total $\boldsymbol{L}$ and $\boldsymbol{S}$ are formed due to strong Hund's coupling prior to the formation of spin-orbit coupled eigenstates. Such process mainly occurs in insulating compounds with localized f states.[1] However, in actinides the spin-orbit (SO) coupling is stronger than the Hund's coupling.[2] Therefore, total angular momentum $\boldsymbol{J}=\boldsymbol{L}+\boldsymbol{S}$ is the good quantum number for this state. In such systems, a SO density wave order in the two-particle channel can arise at some critical value of the coupling constant g, by taking advantage of any instability, such as FS 'nesting' (shown in the main text), even when the time-reversal symmetry remains invariant. The coupling g can be related to some form of 'screened' interorbital Coulomb term. Physically, SO density wave is different from a spin density wave because here spin-flip occurs between two different orbitals without breaking time-reversal symmetry. Therefore, we desire to study a Hamiltonian:

$$H = H_0 + H_{SODW}, \tag{1}$$

where H_0 is the non-interacting part and H_{SODW} is the SO density wave term. In the 'pseudospin' basis introduced in the main text $\hat{\Psi}^{\dagger}(\boldsymbol{k}) = (f^{\dagger}_{\boldsymbol{k},\frac{1}{2},\sigma},\ f^{\dagger}_{\boldsymbol{k},\frac{3}{2},\sigma}, f^{\dagger}_{\boldsymbol{k},\frac{1}{2},\bar{\sigma}},\ f^{\dagger}_{\boldsymbol{k},\frac{3}{2},\bar{\sigma}})$ ($\bar{\sigma}=-\sigma$), the representation of the symmetry operations for URu_2Si_2 system which belong to the D_{4h} symmetry is: time-reversal symmetry $\mathcal{TR}=\mathcal{K}\cdot i\boldsymbol{\sigma}^y\otimes\boldsymbol{\tau}^0$, inversion symmetry $\mathcal{I}=\boldsymbol{\tau}^z\otimes\boldsymbol{\tau}^0$, four-fold rotation symmetry around the z axis $\mathcal{C}_4=\exp\left[i(\pi/4)\boldsymbol{\sigma}^z\otimes\boldsymbol{\tau}^0\right]$ and the two reflection symmetries $\mathcal{P}_{x/y}=i\boldsymbol{\sigma}^{x/y}$ which map $x\rightarrow -x$ (where x is in Γ-X direction) and $y\rightarrow -y$ (where y is orthogonal to Γ-X direction), respectively. Here, $\mathcal{K}$ is complex conjugation operator, and $\boldsymbol{\sigma}^{x,y,z}$ and $\boldsymbol{\tau}^{x,y,z}$ depict the two-dimensional Pauli matrices in the 'pseudospin' and orbital space, respectively where τ^0 is the unitary matrix.

Each symmetry operation transforms the time-reversal invariant f-electron field as:

$$\mathcal{I}\hat{\Psi}(k_x,k_y) \rightarrow -\boldsymbol{\tau}^0\hat{\Psi}(-k_x,-k_y), \tag{2}$$

$$\mathcal{C}_4\hat{\Psi}(k_x,k_y) \rightarrow i\boldsymbol{\tau}^y\hat{\Psi}(-k_y,k_x), \tag{3}$$

$$\mathcal{P}_{x/y}\hat{\Psi}(k_x,k_y) \rightarrow \mp\boldsymbol{\tau}^z\hat{\Psi}(\mp k_x,\pm k_y). \tag{4}$$

These symmetry operations imply that the spin-orbit coupled actinide f-state is an odd-parity wavefunction. Using these symmetry properties, the non-interacting Hamiltonian is deduced in the main text, using standard procedure, see for example Refs. 3, 4.

I. HIDDEN ORDER PARAMETER

Considering the non-interacting FS nesting at $\boldsymbol{Q}_h=(1\pm 0.4,0,0)$ for the hidden order state as demonstrated in the main text, we expand the above-mentioned 'pseudospinor' in the Nambu notation as $\hat{\Psi}(\boldsymbol{k})=\left(\hat{\Psi}(\boldsymbol{k}),\hat{\Psi}(\boldsymbol{k}+\boldsymbol{Q}_h)\right)$. In this notation, the modulated SOC interaction term can be written in general as

$$H_{SODW} = \sum_{\mu\nu} g^{\mu\nu} : \left[\hat{\Psi}^{\dagger}(\boldsymbol{k})\Gamma^{\mu\nu}\hat{\Psi}(\boldsymbol{k}+\boldsymbol{Q}_h)\right]^2 :, \tag{5}$$

where $\mu,\nu\in\{0,x,y,z\}$. The symbol :: represents normal ordering. Here g is the coupling constant discussed latter and $\Gamma^{\mu\nu}=\boldsymbol{\tau}^{\mu}\otimes\boldsymbol{\sigma}^{\nu}$, $\boldsymbol{\tau}$ and $\boldsymbol{\sigma}$ represent Pauli matrices in orbital and spin basis, respectively. Absorbing g and Γ into one term we define the mean-field order parameter

$$M^{\mu\nu} = g^{\mu\nu}(\boldsymbol{k})\left\langle\hat{\Psi}(\boldsymbol{k})^{\dagger}[\boldsymbol{\tau}^{\mu}\otimes\boldsymbol{\sigma}^{\nu}]\hat{\Psi}(\boldsymbol{k}+\boldsymbol{Q}_h)\right\rangle. \tag{6}$$

$$= g^{\mu\nu}(\boldsymbol{k})\left\langle f^{\dagger}_{\boldsymbol{k},\tau,\sigma}[\boldsymbol{\tau}^{\mu}_{\tau\tau'}\otimes\boldsymbol{\sigma}^{\nu}_{\sigma\sigma'}]f_{\boldsymbol{k}+\boldsymbol{Q}_h,\tau',\sigma'}\right\rangle. \tag{7}$$

Here τ,τ' and σ,σ' (not in bold font) are the components of the $\boldsymbol{\tau}^{\mu}$ and $\boldsymbol{\sigma}^{\nu}$ matrices, respectively. Without any loss of generality we fix the spin orientation along z-directions ($\nu=z$). Therefore, we drop the index ν henceforth. Furthermore we define the gap vector as

$$\boldsymbol{b}^{\mu}_{\tau\tau'}(\boldsymbol{k}) = \Delta^{\mu}_{\tau\tau'}(\boldsymbol{k})\boldsymbol{\tau}^{\mu}_{\tau\tau'}, \tag{8}$$

where momentum dependence of the gap function transforms according to the same irreducible representation of the point-group symmetry (g is absorbed in the gap function defined below). With these substitutions, we obtain the final result for the order parameter as

$$M^{\mu} = \left\langle\sum_{\tau\tau'\sigma\sigma'} f^{\dagger}_{\boldsymbol{k},\tau,\sigma}\left[\boldsymbol{b}^{\mu}_{\tau\tau'}(\boldsymbol{k})\boldsymbol{\sigma}^{z}_{\sigma\bar{\sigma}}\right]f_{\boldsymbol{k}+\boldsymbol{Q}_h,\tau',\bar{\sigma}}\right\rangle. \tag{9}$$

For the unidirectional modulation vector $\boldsymbol{Q}_h$, all components of M^{μ} break C_4 rotational symmetry. All the symmetry properties of the order parameters are given in Table I. M^0, M^x and M^z break time-reversal and thus are ruled out as the hidden-order state is arguably does not exhibit any time-reversal symmetry breaking.[5–7]. Although some evidences

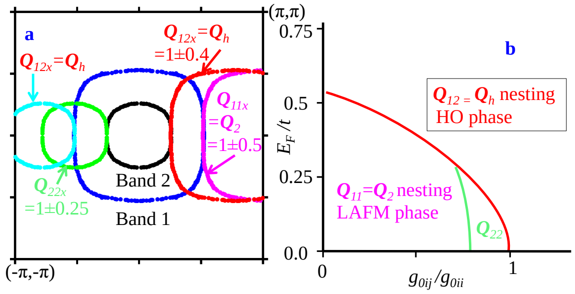

FIG. 1. **a**, Black and blue symbols are *ab-initio* FSs, as shown in Fig. 1 of main text, but plotted here only two relevant bands. Two large square FSs for band 1 are shifted along the $x-$direction by nesting vectors $\boldsymbol{Q}_{11}=\boldsymbol{Q}_2$ and $\boldsymbol{Q}_{12}$, while the two small square FSs for band 2 are shifted along $-x$ direction. Good nestings for all three vectors are observed, but the intraband one are protected by strong SOC, where $\boldsymbol{Q}_{12}=\boldsymbol{Q}_h$ is the hidden order nesting that opens a gap. **b**, Phase diagram as a function of intra-orbital ($g_{0ij}=g_{012}=g_{021}$) and inter-orbital coupling constant ($g_{0ii}=g_{011}=g_{022}$) and nearest neighbor hopping (t) with respect to the Fermi energy (E_F) for all thee nesting instabilities. We find that for smaller inter-orbital coupling than the intra-orbital one, the hidden order parameter arises if E_F/t ration is large. With large t (proportional to pressure), the LMAF phase at the commensurate nesting wins. This result is consistent, at least, in qualitative level, with the phase diagram of URu_2Si_2.[8]

for the time-reversal symmetry breaking are also present, but it is not well established if the measurements are done in single crystal where the time-reversal symmetry breaking LMAF state and time-reversal symmetry invariant HO state are not mixed. In microscopic sense, LMAF and HO state are separated by phase transition, and thus both phases cannot inherit same broken symmetry.

	M^0	M^x	M^y	M^z
$\mathcal{TR}$	-	-	+	-
$\mathcal{C}_4$	-	-	-	-
$\mathcal{I}$	+	+	+	+
$\mathcal{P}_{x/y}$	+	-	-	+

TABLE I. Symmetry properties of various density wave parameters. '+' ('-') represent 'even' ('odd') parity of the order parameter under the corresponding symmetry operations.

On the other hand, M_2 is even under time-reversal but odd under parity, and thus adds a mass term to the Hamiltonian which opens a gap at the nested portion of the Fermi surface. This is the term that represents modulated spin-orbit coupling. A trivial check can be performed by explicitly writing down the the spin-orbit coupling term $\sum_i(-1)^{i_x}L_{i,z}S_{i,z}\propto f^{\dagger}_{i,+,\sigma}f_{i,+,\sigma'}-f^{\dagger}_{i,-,\sigma}f_{i,-,\sigma'}=2if^{\dagger}_{i,\frac{1}{2},\sigma}f_{i,\frac{3}{2},\sigma'}+h.c.$, where we substituted $f_{i,\pm,\sigma}=(f_{i,\frac{1}{2},\sigma}\pm if_{i,\frac{3}{2},\sigma})$.

With this definition, the total Hamiltonian in the reduced Brillouin zone can be written as

$$
\begin{aligned}
H = & \sum_{\boldsymbol{k},\tau,\tau',\sigma}{}^{'} \Big(h_{\tau\tau'}(\boldsymbol{k}) f^{\dagger}_{\boldsymbol{k},\tau,\sigma} f_{\boldsymbol{k},\tau',\bar{\sigma}} \\
& + h_{\tau\tau'}(\boldsymbol{k}+\boldsymbol{Q}_h) f^{\dagger}_{\boldsymbol{k}+\boldsymbol{Q}_h,\tau,\sigma} f_{\boldsymbol{k}+\boldsymbol{Q}_h,\tau',\bar{\sigma}} \Big) \\
& - \sum_{\boldsymbol{k},\mu,\tau,\tau',\sigma}{}^{'} \Big(M^{\mu}(\boldsymbol{k}) f^{\dagger}_{\boldsymbol{k},\tau,\sigma} [\boldsymbol{\tau}^{\mu}_{\tau\tau'}\otimes\boldsymbol{\sigma}^{\nu}_{\sigma\sigma'}] f_{\boldsymbol{k}+\boldsymbol{Q}_h,\tau',\sigma'} \\
& + M^{\mu}(\boldsymbol{k}+\boldsymbol{Q}_h) f^{\dagger}_{\boldsymbol{k}+\boldsymbol{Q}_h,\tau,\sigma} [\boldsymbol{\tau}^{\mu}_{\tau\tau'}\otimes\boldsymbol{\sigma}^{\nu}_{\sigma\sigma'}] f_{\boldsymbol{k},\tau',\sigma'} \Big) \\
& + \sum_{\boldsymbol{k},\mu} M^{\mu}(\boldsymbol{k}) M^{\mu}(\boldsymbol{k}+\boldsymbol{Q}_h)/g^{\mu}. \qquad (10)
\end{aligned}
$$

Where h is the non-interacting part of the Hamiltonian defined in Eq. 2 of the main text. The prime over a summation implies that the summation is performed in the reduced Brillouin zone. Diagonalizing the above Hamiltonian, we deduce the quasiparticle states are: $E^{\tau\sigma}_{\boldsymbol{k},\nu}=\xi^{\tau\sigma}_{\boldsymbol{k}+}+\nu E^{\tau\sigma}_{\boldsymbol{k},0}$, and $E^{\tau\sigma}_{\boldsymbol{k},0}=\sqrt{(\xi^{\tau\sigma}_{\boldsymbol{k}-})^2+|\Delta_{\tau\tau'}|^2}$, where $\xi^{\tau\sigma}_{\boldsymbol{k}\pm}=(E^{\tau\sigma}_{\boldsymbol{k}}\pm E^{\tau\sigma}_{\boldsymbol{k}+\boldsymbol{Q}})/2$, and

$$
E^{\tau\sigma}(\boldsymbol{k})=\epsilon(\boldsymbol{k})+\tau\sqrt{\sum_{\mu}|\boldsymbol{d}^{\mu}_{12}(\mathbf{k})|^2}+\sigma\sqrt{\sum_{\mu}|\boldsymbol{d}^{\mu}_{11}(\boldsymbol{k})|^2} \qquad (11)
$$

from Eq. 3 of the main text. $\nu,\nu'=\pm$. The corresponding coherence factors are

$$
u^{\tau\sigma}_{\boldsymbol{k}}(v^{\tau\sigma}_{\boldsymbol{k}})=\frac{1}{2}\left(1\pm\frac{\xi^{\tau\sigma}_{\boldsymbol{k}-}}{E^{\tau\sigma}_{\boldsymbol{k},0}}\right). \qquad (12)
$$

II. SELF-CONSISTENT GAP EQUATION

We can easily derive the self-consistent gap equation from Eq. 9 using Bogolyubov treatment. We substitute the fermion operator f in terms of a Bogolyubov operators as

$$
\begin{aligned}
f_{\boldsymbol{k},\tau\sigma} &= u^{\tau\sigma}_{\boldsymbol{k}}\gamma_{\boldsymbol{k},\tau\sigma}-v^{\tau\sigma}_{\boldsymbol{k}}\gamma_{\boldsymbol{k}+\boldsymbol{Q}_h,\tau\sigma} \\
f_{\boldsymbol{k}+\boldsymbol{Q}_h,\tau\sigma} &= v^{\tau\sigma}_{\boldsymbol{k}}\gamma_{\boldsymbol{k},\tau\sigma}+u^{\tau\sigma}_{\boldsymbol{k}}\gamma_{\boldsymbol{k}+\boldsymbol{Q}_h,\tau\sigma}. \qquad (13)
\end{aligned}
$$

From Eqs. 7, 8, and 9, we get the gap function as

$$
\begin{aligned}
\Delta^{\mu}_{\tau\tau'} &= g^{\mu}_{\tau\tau'}\sum_{\boldsymbol{k},\sigma}\left\langle f^{\dagger}_{\boldsymbol{k}+\boldsymbol{Q}_h,\tau\sigma}f_{\boldsymbol{k},\tau'\bar{\sigma}}\right\rangle, \qquad (14) \\
&= g^{\mu}_{\tau\tau'}\sum_{\boldsymbol{k},\sigma}u^{\tau\sigma}_{\boldsymbol{k}}v^{\tau'\bar{\sigma}}_{\boldsymbol{k}}\left[\left\langle\gamma^{\dagger}_{\boldsymbol{k},\tau\sigma}\gamma_{\boldsymbol{k},\tau'\bar{\sigma}}\right\rangle\right. \\
&\quad \left.-\left\langle\gamma^{\dagger}_{\boldsymbol{k}+\boldsymbol{Q}_h,\tau}\gamma_{\boldsymbol{k}+\boldsymbol{Q}_h,\tau'\bar{\sigma}}\right\rangle\right], \\
&= g^{\mu}_{\tau\tau'}\sum_{\boldsymbol{k},\sigma}u^{\tau\sigma}_{\boldsymbol{k}}v^{\tau'\bar{\sigma}}_{\boldsymbol{k}}\left[n^{+}_{f,\tau\sigma}(\boldsymbol{k})-n^{-}_{f,\tau'\bar{\sigma}}(\boldsymbol{k})\right], \\
&= \frac{1}{2}g^{\mu}_{\tau\tau'}\sum_{\boldsymbol{k},\sigma}\frac{\Delta^{\mu}_{\tau\tau'}(\boldsymbol{k})}{E^{\tau\sigma}_{\boldsymbol{k}}}\left[n^{+}_{f,\tau\sigma}(\boldsymbol{k})-n^{-}_{f,\tau'\bar{\sigma}}(\boldsymbol{k})\right]. \qquad (15)
\end{aligned}
$$

Here we have substituted $\langle\gamma^{\dagger}_{\boldsymbol{k},\tau\sigma}\gamma_{\boldsymbol{k},\tau\sigma}\rangle = n^{\nu}_{f,\tau\sigma}(\boldsymbol{k})$, where n is the Fermi function. We find that $g_{12} = 27$ meV gives Δ =5 meV in consistent with experiments. For this temperature independent value of g_{12}, we obtain $T_h = 22$K, which is higher than the experimental value of $T_h = 17.5$ meV. Possible reason for overestimating the value of T_h are the neglect of quantum fluctuation, disorder which can reduce its value. In this context, it can be noted that recently, a 'pseudogap' phase upto 20 K is marked from experimental features,[17] which arguably suggests that there are indeed fluctuations present above T_h.

III. SPIN-ORBIT CORRELATION FUNCTION

The non-interacting single-particle Green's function for the Hamiltonian given in Eq. 1 above is defined as $G_{\tau\tau'\sigma\sigma'}(\boldsymbol{k}, i\omega_n) = -1/\beta\sum_n\langle T_{\mathcal{T}} f_{\boldsymbol{k}\tau\sigma}(\mathcal{T}) f^{\dagger}_{\boldsymbol{k}\tau'\sigma'}(0)\rangle e^{i\omega_n\mathcal{T}}$. Here $\mathcal{T}$ is the imaginary time, $f_{\boldsymbol{k}\tau\sigma}(\mathcal{T})$ is the imaginary time evolution of the fermionic operator $f_{\boldsymbol{k}\tau\sigma}$, n is the fermionic Matsubara frequency, and $\beta = 1/k_BT$, where k_B is Boltzmann constant. $T_{\mathcal{T}}$ gives normal time-ordering. The anomalous part of the Green's function is $F_{\tau\tau'\sigma\sigma'}(\boldsymbol{k}, i\omega_n) = -\frac{1}{\beta}\sum_n\left\langle T_{\mathcal{T}} f_{\boldsymbol{k}\tau\sigma}(\mathcal{T}) f^{\dagger}_{(\boldsymbol{k}+\boldsymbol{Q})\tau'\sigma'}(0)\right\rangle e^{i\omega_n\mathcal{T}}$. By Fourier transforming in the Matsubara frequency space, we obtain the explicit forms of two Green's functions as

$$G_{\tau\tau'\sigma\sigma'}(\boldsymbol{k}, i\omega_n) = \sum_{\nu} R^{\nu}_{\tau\tau'\sigma\sigma'}(\boldsymbol{k}) \times\left(\frac{(u^{\tau\sigma}_{\boldsymbol{k}})^2}{i\omega_n - E^{\tau\sigma}_{\boldsymbol{k},\nu}} + \frac{(v^{\tau'\sigma'}_{\boldsymbol{k}})^2}{i\omega_n - E^{\tau'\sigma'}_{\boldsymbol{k},\nu}}\right) \quad (16)$$

$$F_{\tau\tau'\sigma\sigma'}(\boldsymbol{k}, i\omega_n) = \sum_{\nu} R^{\nu}_{\tau\tau'\sigma\sigma'}(\boldsymbol{k}) u^{\tau\sigma}_{\boldsymbol{k}} v^{\tau'\sigma'}_{\boldsymbol{k}} \times\left(\frac{1}{i\omega_n - E^{\tau\sigma}_{\boldsymbol{k},\nu}} - \frac{1}{i\omega_n - E^{\tau'\sigma'}_{\boldsymbol{k},\nu}}\right) \quad (17)$$

The orbital overlap matrix-element $R^{\nu}_{\tau\tau'\sigma\sigma'}(\boldsymbol{k}) = \psi^{\nu}_{\tau\sigma}(\boldsymbol{k})\psi^{\nu\dagger}_{\tau'\sigma'}(\boldsymbol{k})$, where ψ is the eigenvector of the non-interacting Hamiltonian, projects the Green's function from band basis to the orbital one. However, to simplify our calculation, we assume that each block state $E^{\tau\sigma}$ corresponds to each orbital which makes $R = 1$. By taking analytical continuation of the Matsubara frequency to the real frequency in the above Green's function, it is easy to show that the gap function Δ can be obtained by averaging the anomalous Green's function.

The general form of the polarization vector is

$$J^z_{\tau\tau'\sigma\sigma'}(\boldsymbol{q}, \mathcal{T}) = \sum_{\boldsymbol{k}} f^{\dagger}_{\boldsymbol{k},\tau,\sigma}(\mathcal{T})\boldsymbol{\tau}^{\mu}_{\tau\tau'}\boldsymbol{\sigma}^{z}_{\sigma\sigma'} f_{\boldsymbol{k}+\boldsymbol{q},\tau',\sigma'}(\mathcal{T}). \quad (18)$$

To simplify the notations, we define composite indices $\alpha, \beta = \tau\tau'\sigma\sigma'$, in which the correlation function of J^z_{α} vector can now be defined as

$$\chi^{zz}_{\alpha\beta}(\boldsymbol{q}, \mathcal{T}) = \frac{1}{N}\left\langle T_{\mathcal{T}} J^z_{\alpha}(\boldsymbol{q}, \mathcal{T}) J^z_{\beta}(-\boldsymbol{q}, 0)\right\rangle. \quad (19)$$

Substituting J^z_{α} and then applying standard Wick's decomposition to the electron bracket[9] yields,

$$\chi^{zz}_{\alpha\beta}(\boldsymbol{q}, ip_m) = -\frac{1}{N}\sum_{\boldsymbol{k},n}[G_{\alpha}(\boldsymbol{k}, i\omega_n)G_{\beta}(\boldsymbol{k}+\boldsymbol{q}, i\omega_n + ip_m) - F_{\alpha}(\boldsymbol{k}, i\omega_n)F_{\beta}(\boldsymbol{k}+\boldsymbol{q}, i\omega_n + ip_m)\rangle]. \quad (20)$$

$$= \frac{1}{N}\sum_{\boldsymbol{k}} S_{\alpha\beta}(\boldsymbol{k}, \boldsymbol{q}) \quad (21)$$

$$\times\Big[\left(u_{\boldsymbol{k}}u_{\boldsymbol{k}+\boldsymbol{q}} + v_{\boldsymbol{k}}v_{\boldsymbol{k}+\boldsymbol{q}}\right)^2\left(\chi^{++} + \chi^{--}\right) + \left(u_{\boldsymbol{k}}v_{\boldsymbol{k}+\boldsymbol{q}} - v_{\boldsymbol{k}}u_{\boldsymbol{k}_{\boldsymbol{q}}}\right)^2\left(\chi^{+-} + \chi^{-+}\right)\Big] \quad (22)$$

Here $S_{\alpha\beta}(\boldsymbol{k}, \boldsymbol{q}) = R_{\alpha}(\boldsymbol{k})R_{\beta}(\boldsymbol{k}+\boldsymbol{q})$ is the matrix-element term which projects the susceptibility from the band representation to the orbital one. $\chi^{\nu\nu'}$ are the fermionic oscillator terms in the band space $\nu, \nu' = \pm$:

$$\chi^{\nu\nu'} = -\frac{n^{\nu}_f(\boldsymbol{k}) - n^{\nu'}_f(\boldsymbol{k}+\boldsymbol{q})}{ip_m - E^{\nu}_{\boldsymbol{k}} - E^{\nu'}_{\boldsymbol{k}+\boldsymbol{q}}}. \quad (23)$$

The momentum, energy, α indices on both sides of the above equation are implicit.

A. Hidden order instability

The divergence in the real-part of the $\chi_{\alpha\beta}(\boldsymbol{q}, \omega = 0)$ indicates an instability due to the FS nestings between two orbitals α and β (in the explicit notation χ_{11} means χ_{1111}, etc). The two intraband nestings at Q_{11} and Q_{22} can give rise to spin-density wave or antiferromagnetism, if the time-reversal symmetry is broken. We believe that Q_{11} is responsible for the LMAF phase. Our present interest is the interband one at Q_{12}, shown in supplementary Fig. 1(*A*) which happens at $(1 \pm 0.4, 0, 0)$. Since in this case, both orbital and spin flip together, the time-reversal symmetry remains intact.

The corresponding critical value of the coupling constants $g_{\alpha\beta}$ at which a gap opening or an order parameter develops can be evaluated within random-phase approximation (RPA). The stoner criterion for an instable state implies that $1-\chi'_{\alpha\beta}(\boldsymbol{Q}_{\alpha\beta}, 0)g_{\alpha\beta} \geq 0$, or $g_{\alpha\beta} \leq 1/\chi'_{\alpha\beta}(\boldsymbol{Q}_{\alpha\beta}, 0)$. Looking at the FS areas for each band, shown in supplementary Fig. 1**a**, we immediately see that $1/\chi'_{11}(\boldsymbol{Q}_{11}, 0) < 1/\chi'_{22}(\boldsymbol{Q}_{22}, 0) < 1/\chi'_{12}(\boldsymbol{Q}_{12}, 0)$, leading to a phase diagram shown in supplementary Fig. 1(*B*). The present calculation does not incorporate the possible coexistence state between different phases. The phase diagram implies that there is a considerably large parameter space, where the $\boldsymbol{Q}_{12} = \boldsymbol{Q}_h$ nesting dominates. We have not considered all possible phases except M^y for Q_{12} nestings discussed in Eq. 9 above, because constrained by the symmetry arguments given in Table I, others render gapless state.

B. Neutron mode at the Hidden order state

Eqs. 22 and 23, imply that the hidden order transition accompanies an inelastic neutron scattering mode with en-

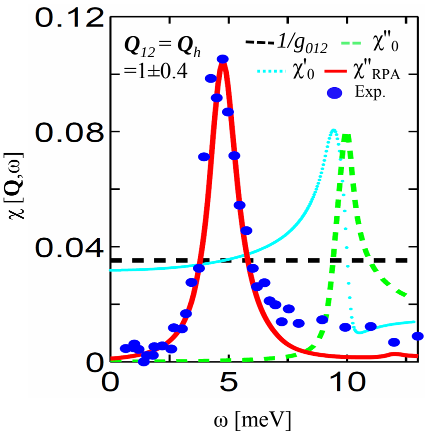

FIG. 2. The real and imaginary part of the bare susceptibility calculated from Eqs. 22-23 in the hidden order state. The red line gives the INS mode obtained using RPA calculation at $g_{12} = 28.4$ meV whose inverse is shown by horizontal dashed line. The RPA value has a huge peak (resonance) at ω_Q =4.7 meV when $\chi'(\boldsymbol{Q}_h, \omega_Q) = 1/g_{12}$, and we have rescaled its intensity arbitrarily to fit it into the same figure with the bare values for comparison. The obtained resonance is in good agreement with experiment shown by symbols.[11]

hanced intensity at Q_h whose energy scale is approximately given by $\chi''(\boldsymbol{Q}_h, \omega) \sim C\delta(\omega - E^1_{\boldsymbol{k}} - E^2_{\boldsymbol{k}+\boldsymbol{Q}_h}) \approx C\delta(\omega - |\Delta(\boldsymbol{k})| - |\Delta(\boldsymbol{k} + \boldsymbol{Q}_h)|)$. C is the prefactor which has to be evaluated rigorously, but it does not contribute to the peak position in bare χ''. Here we have substituted the condition that non-interacting bands are nested on the FS at Q_h such as $E^\nu_{\boldsymbol{k}} = |\Delta(\boldsymbol{k})|$ ≈5 meV. The RPA correction shifts the resonance energy which depends on the value of coupling constant. At $g_{12} = 28.4$ meV, the $1/g_{12}$ line cuts twice to $\chi'(\boldsymbol{Q}_h, \omega)$, however, the resonance is stronger at the energy where the broadening function coming from χ'' is weaker. Therefore, the strong intensity or a resonance occurs at $\omega_Q \sim 4.7$ meV, see Fig. 2. INS measurements have observed this resonance at the incommensurate nesting vector $Q = (1.4, 0, 0)$.[11–13] However, one have to be careful to directly compare our result with this data. Because, in the present case, we expect a mode which does not break time-reversal symmetry. Therefore, in order to observe this mode, one requires to perform a polarized INS measurement.

On the same reasoning, INS should see more resonances at Q_{11} and Q_{22} even in the non-polarized condition. However, the energy scale and intensity of those modes will depend on the location of the phase diagram of URu_2Si_2 where the experiment is performed. As we argued earlier, Q_{11} is most likely responsible for the LMAF phase, therefore, we can expect a mode at twice of the corresponding gap opening. The peak in the INS spectra at the commensurate vector is also observed at a much lower in energy, however, the peak is much broader than the resonance peak observed at the incommensurate vector.[11, 13]

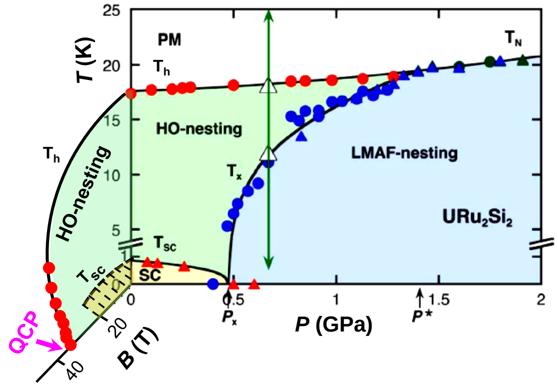

FIG. 3. Proposed phase diagram of URu_2Si_2 along the magnetic field ($\boldsymbol{B}$) direction. The data along pressure $(P) - T$ plane is taken from Ref. 8. The symbols along $B - T$ axes are extracted from Ref. 18. A QCP at $T = 0$ along the field directions is expected from our theory, and also observed in experiment.[18] Deducing the phase diagram for SC and other possible phases that may arise above the QCP is beyond the scope of the present calculation.

IV. QUANTUM CRITICAL POINT

As mentioned in the main text, any $\mathcal{TR}$ breaking perturbation such as magnetic field will destroy the $\mathcal{TR}$ invariant HO phase. As the HO state incipiently is a spontaneously broken symmetry, it will exhibit a second order phase transition along the field axis. In what follows we expect to obtain a quantum critical point (QCP) at $T = 0$, as extensively proposed to be associated with any second order phase transition,[19] along the magnetic field axis. We find the critical field to be B ≈38 T, whereas the experimental data[18] indicates that the QCP resides around $B \sim 34$ T. The present model can not deduce the phase diagram for the superconducting (SC) state, possibly intervening the HO state, or any other phases that may arise above the QCP.[18] More experimental data and theoretical modelling are required to understand the details of this regime of the phase diagram.

[1] Dzero, M., Sun, K., Galitski, V., Coleman, P. Topological Kondo Insulators. *Phys. Rev. Lett.* **104**, 106408 (2010).
[2] Hotto, T., Ueda, K. Construction of a microscopic model for f-electron systems on the basis of a $j - j$ coupling scheme. *Phys. Rev. B* **67**, 104518 (2003).
[3] Bernevig, B. A., Hughes, T. L., Zhang, S. C. Quantum spin Hall effect and topological phase transition in HgTe quantum wells. *Science* **314**, 1757 (2006).
[4] Qi, X. L., Hughes, T. L., Zhang, S. C. Topological field theory of time-reversal invariant insulators. *Phys. Rev. B* **78**,195424 (2008).
[5] Haule, K., Kotliar, G. Arrested Kondo effect and hidden order in URu_2Si_2. *Nat. Phys.* **5**, 796-799 (2009).
[6] Dubi, Y., Balatsky, A. V. Hybridization Wave as the Hidden Order in URu_2Si_2. *Phys. Rev. Lett.* **106**, 086401 (2011).

[7] Santini, P. Behavior of URu_2Si_2 in an applied magnetic field. *Phys. Rev. B* **57**, 5191-5199 (1998).

[8] Hassinger, E., Knebel, G., Izawa, K., Lejay, P., Salce, B., Flouquet, J. Temperature-pressure phase diagram of URu_2Si_2 from resistivity measurements and ac calorimetry: Hidden order and Fermi-surface nesting. *Phys. Rev. B* **77**, 115117 (2008).

[9] Mahan, G. D. Many-Particle Physics, *Springer; 3nd edition* (2000).

[10] Broholm, C. *et al.* Magnetic excitations and ordering in the heavy-electron superconductor URu_2Si_2. *Phys. Rev. B* **43**, 12809 (1991).

[11] Bourdarot, F. *et al. J. Phys. Soc. Jap.* **79**, 064719 (2010).

[12] Wiebe, C. R. *et al.* Gapped itinerant spin excitations account for missing entropy in the hidden-order state of URu_2Si_2. *Nat. Phys.* **3**, 96 (2007).

[13] Niklowitz, P. G. *et al.* Role of commensurate and incommensurate low-energy excitations in the paramagnetic to hidden-order transition of URu2Si2 *Preprint avaiable at http://arxiv.org/abs/1110.5599* (2011).

[14] Chandra, P. *et al.* Hidden orbital order in the heavy fermion metal URu_2Si_2. *Nature* **417**, 831-834 (2002).

[15] Pathria RK (1996) Statistical Mechanics, (*Butterworth-Heinemann publishers, 2nd Edition*, (1996).

[16] Palstra, T. T. M. *et al.* Superconducting and magnetic transitions in the heavy-fermion system URu_2Si_2. *Phys. Rev. Lett.* **55**, 2727 (1985).

[17] Haraldsen, J. T. *et al.* Hidden-order pseudogap in URu_2Si_2. *Phys. Rev. B* **84**, 214410 (2011).

[18] Kim, K. H. *et al.* Magnetic-Field-Induced Quantum Critical Point and Competing Order Parameters in URu_2Si_2. *Phys. Rev. Lett.* **91**, 256401 (2003).

[19] Sachdev. S. Quantum phase transitions, *Cambridge University Press, Cambridge U.K.* (1999).
